# Hyalodendrins A and B, New Decalin-Type Tetramic Acid Larvicides from the Endophytic Fungus *Hyalodendriella* sp. Ponipodef12

**DOI:** 10.3390/molecules25010114

**Published:** 2019-12-27

**Authors:** Ziling Mao, Weixuan Wang, Ruixue Su, Gan Gu, Zhi Long Liu, Daowan Lai, Ligang Zhou

**Affiliations:** 1 Department of Plant Pathology, College of Plant Protection, China Agricultural University, Beijing 100193, China; maoziling2011@163.com (Z.M.); wwxcau@163.com (W.W.); 18811655616@163.com (R.S.); ggboy@cau.edu.cn (G.G.); 2 Department of Entomology, College of Plant Protection, China Agricultural University, Beijing 100193, China; zhilongliu@cau.edu.cn

**Keywords:** tetramic acid, hyalodendrin, plant endophytic fungus, *Hyalodendriella* sp., structural elucidation, larvicidal activity

## Abstract

Two new decalin/tetramic acid hybrid metabolites, hyalodendrins A (**1**) and B (**2**) were isolated from plant endophytic fungus *Hyalodendriella* sp. Ponipodef12. The structures of the new compounds were elucidated by analysis of the spectroscopic data, including NMR, HRMS and ECD, and by chemical conversion. Compounds **1** and **2** were phomasetin analogues, and both showed potent larvicidal activity against the fourth-instar larvae of *Aedes aegypti* with the median lethal dose (LC_50_) values of 10.31 and 5.93 μg/mL, respectively.

## 1. Introduction

Mosquitoes are notorious for transmitting various human diseases, such as malaria, dengue fever, yellow fever and filariasis, throughout the world [1]. The mosquito *Aedes aegypti* L. is a competent vector for arboviruses including yellow fever, dengue, chikungunya and Zika [2]. Insecticides are currently used to control this vector, such as pyrethroid, organochlorine, organophosphates and insect growth regulators [1,3]. However, frequent use of these synthetic insecticides not only leads to undesirable effects on the environment, but also increases resistance to insecticides [1,4]. Thus, new larvicides to mosquitoes with different scaffolds are urgently needed.

**Figure 1:**
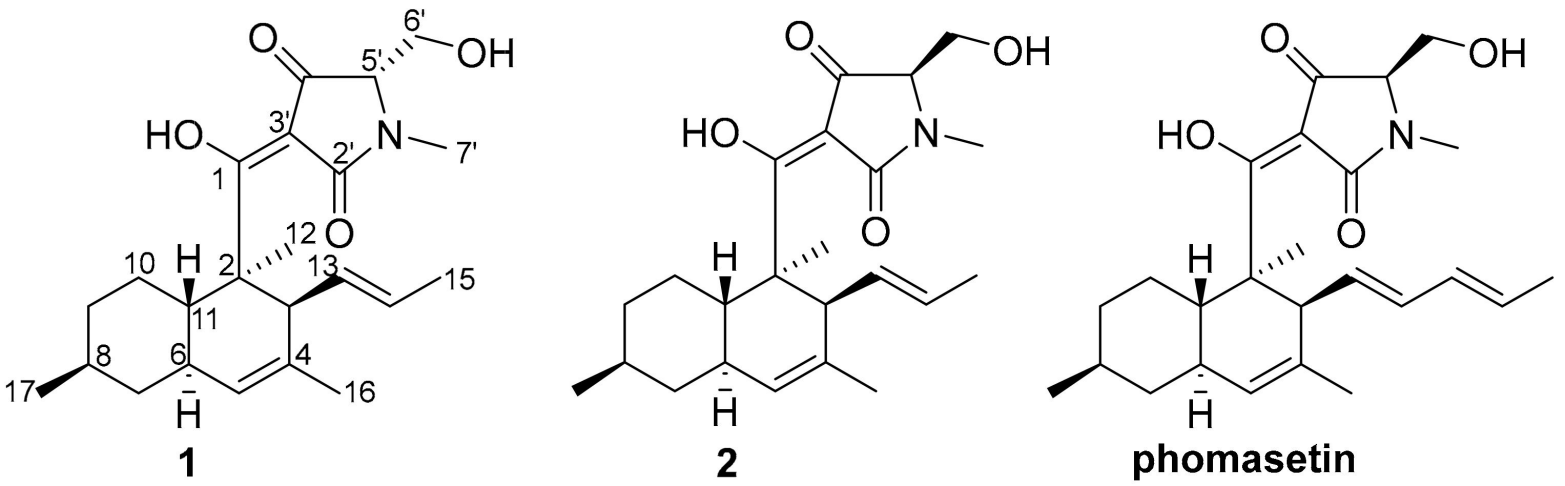
Structures of the isolated compounds **1** and **2**, and phomasetin.

Plant endophytic fungi have been considered as an abundant and important microbial resource for discovering novel larvicidal compounds [5,6,7], such as palmarumycins C_8_ and B_6_ from *Berkleasmium* sp. [8]. *Hyalodendriella* sp. Ponipodef12 was isolated as an endophytic fungus from the hybrid poplar of *Populus deltoides* × *P. nigra* [9]. This endophytic fungus was known to produce benzo-α-pyrones, dibenzo-α-pyrones [10,11,12,13] and 14-membered resorcylic acid lactones [14], and in our previous investigations, among them dibenzo-α-pyrones showed larvicidal activities [10]. Nevertheless, *Hyalodendriella* sp. Ponipodef12 was re-fermented in rice media on a large scale, as some unknown metabolites with a different structure type, were noticed in the larvicidal fraction, but failed to be purified previously, due to the limited amount available. This fraction was pooled and subjected to column chromatography, which resulted in the isolation of two new larvicidal metabolites, namely hyalodendrins A (**1**) and B (**2**) (Figure 1). Details of the isolation, structural elucidation and larvicidal activity of the two compounds are reported herein.

## 2. Results and Discussion

### 2.1. Structural Elucidation of the Compounds

**Figure 2:**
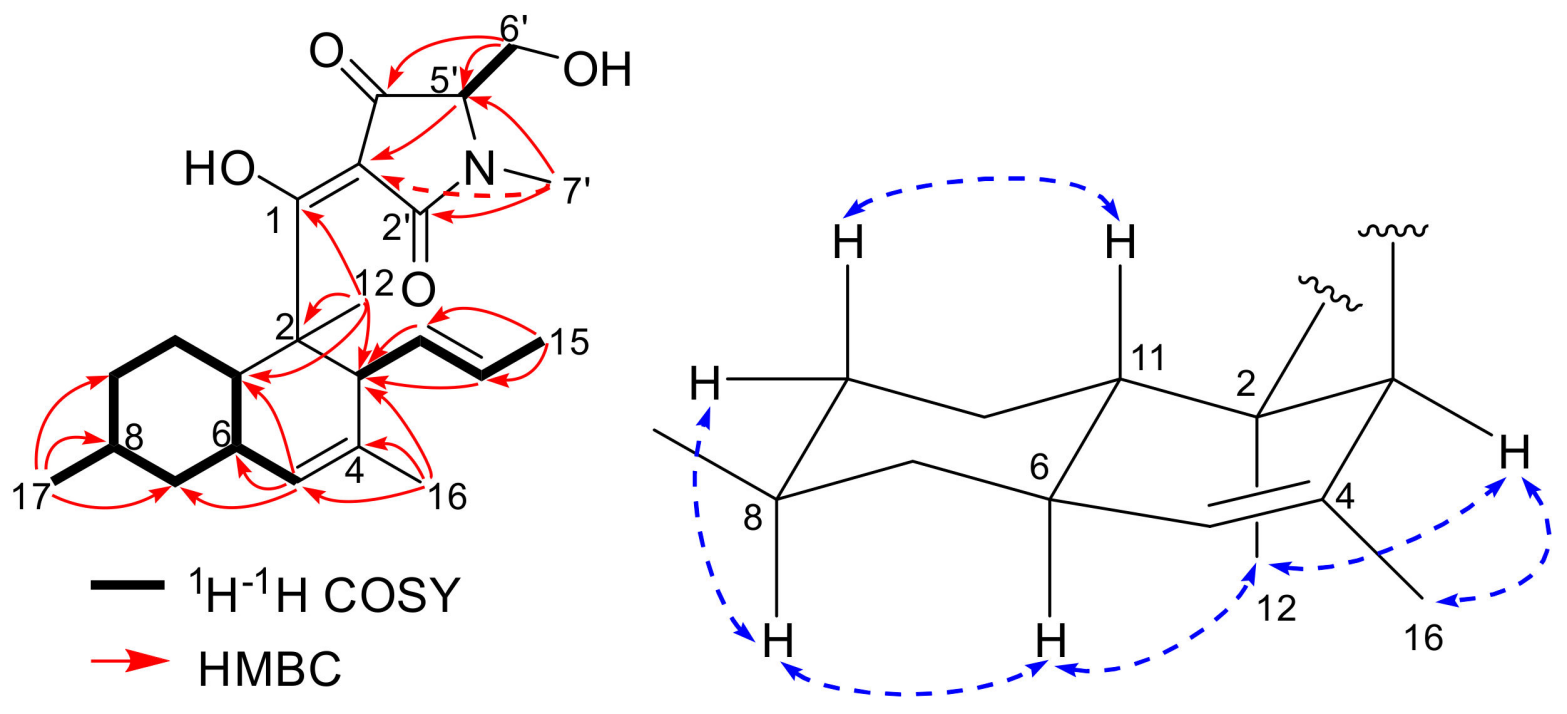
Selected ^1^H‒^1^H COSY, HMBC (**left**) and ROESY (H→H, **right**) correlations of **1**.

Hyalodendrin A (**1**) was isolated as a colorless amorphous powder. It showed a prominent pseudomolecular peak at *m*/*z* 388.24854 [M + H]^+^ in the positive high resolution electrospray ionization mass spectrometry (HRESIMS, Figure S2), indicating its molecular formula as C_23_H_33_NO_4_ with eight degrees of unsaturation. The UV spectrum (Figure S1) showed maximal absorptions at 205, 235 and 294 nm, which were similar to those of phomasetin [15]. The NMR data (Table 1) of **1** were also similar to those of phomasetin [15], except that two sp^2^-carbon signals were missing in **1**, suggesting it was an analogue of phomasetin but with one less double bond. This agreed with the molecular formula of **1** being one C_2_H_2_ unit less than that of phomasetin. Careful examination of the 2D NMR spectra (Figures S5–S8) revealed that the differences were ascribed to the chain attached to C-3, in which a propenyl was found in **1** instead of a penta-1,3-dienyl in phomasetin. This was confirmed by the HMBC experiment, as the correlations were found from Me-15 (*δ*_H_ 1.53, d) to C-13 (*δ*_C_ 130.6) and C-14 (*δ*_C_ 127.7); and from H-14 (*δ*_H_ 5.23, dq) and H-13 (*δ*_H_ 5.11, m) to C-3 (*δ*_C_ 49.4) (Figure 2). Meanwhile, the ^1^H–^1^H COSY spectrum (Figure S5) showed the correlations from Me-15 to H-14, H-14 to H-13, and H-13 to H-3 (*δ*_H_ 3.06, m) (Figure 2), which corroborated the proposed structure.

**Figure 3:**
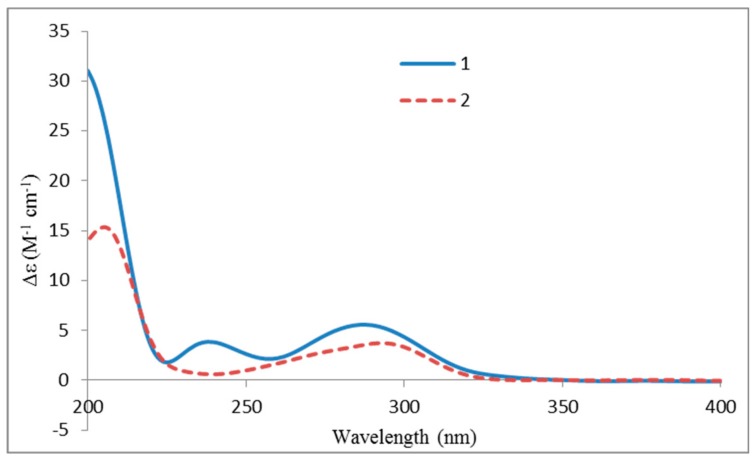
ECD spectra of **1** and **2**.

The relative configuration of the decalin part was found to be the same as that of phomasetin as deduced from the ROESY spectra (Figure S8). The ROESY correlations observed between H-8 (*δ*_H_ 1.49, m)/H-6 (*δ*_H_ 1.82, m), H-6/Me-12 (*δ*_H_ 1.42, s), Me-12/H-3 (*δ*_H_ 3.06, m), H-8/H-9eq (*δ*_H_ 1.75, m) and H-3/Me-16 (*δ*_H_ 1.60, s) revealed that these protons directed to the same face (α), while H-11 (δ_H_ 1.66, m) was found to be correlated with H-9ax (δ_H_ 1.11, m), thus they were β-oriented (Figure 2). This revealed a trans-decalin system in **1**, similar to that of phomasetin and equisetin [15], in which the tetramic acid moiety was equatorial, while the propenyl group was axial. It was reported that the circular dichroism (CD) spectra of phomasetin and its analogues are predominated by the decalin stereochemistry [15]. As the CD spectrum (Figure 3) of **1** was similar to those of phomasetin [15], the same absolute configuration was deduced for the decalin part. However, the configuration of the tetramic acid moiety was deduced to be opposite to that of phomasetin (vide infra), by scrutiny of the NMR data.

**Scheme 1:**
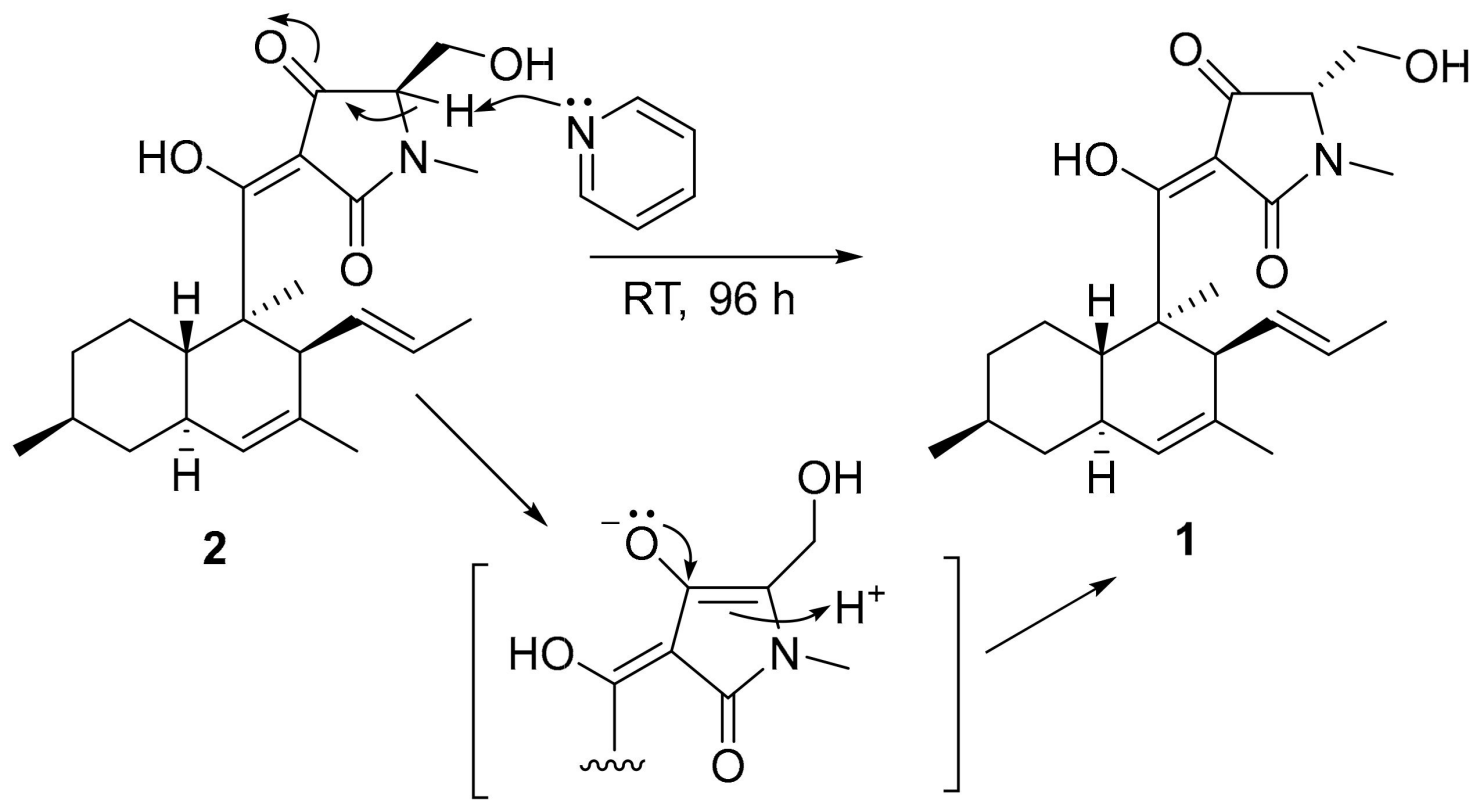
Epimerization of **2** to **1** in pyridine and the possible mechanism.

**Figure 4:**
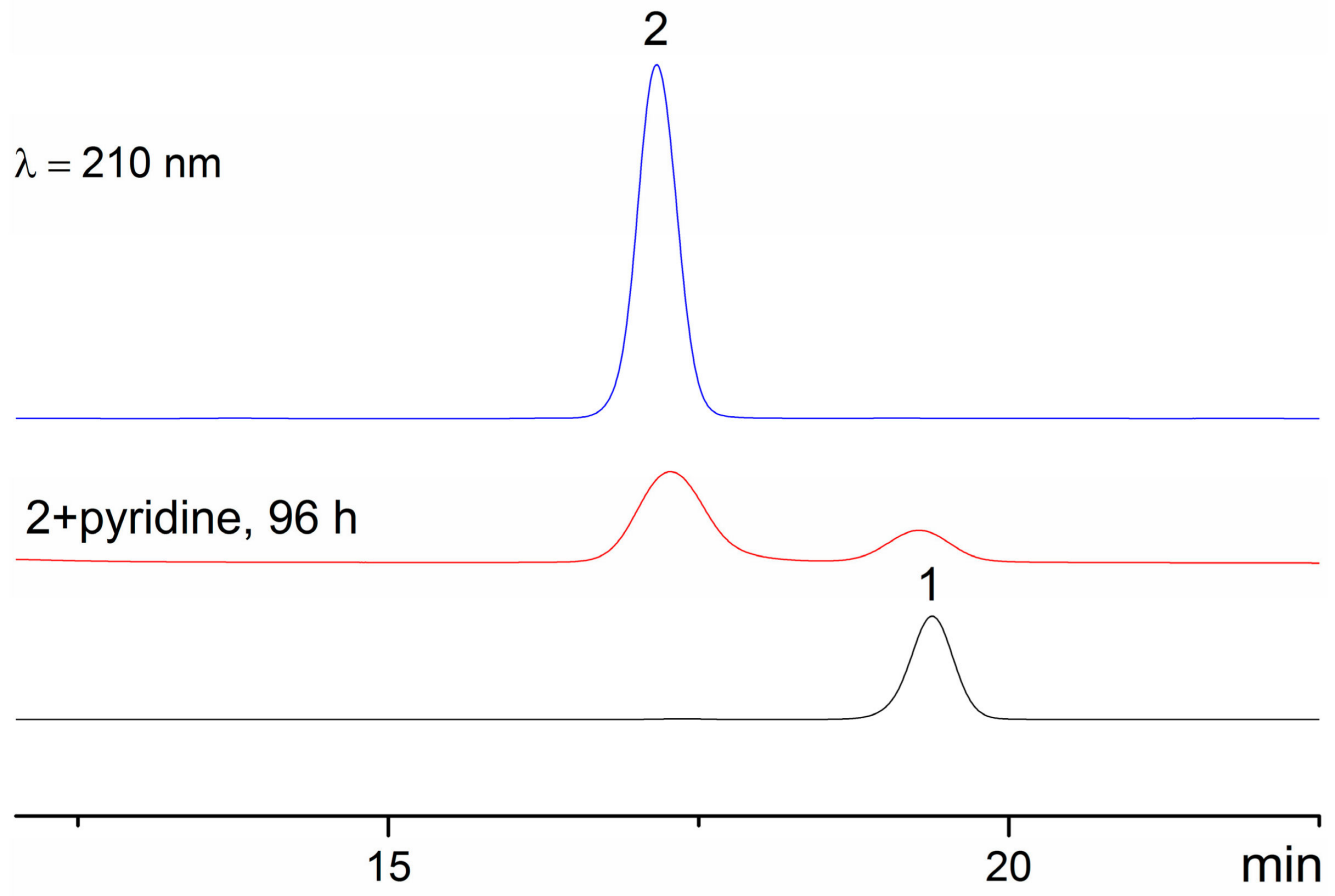
HPLC chromatograms of the epimerized product of **2** in pyridine (retention time, 96 h), and the standards of **1** and **2**.

Hyalodendrin B (**2**) was isolated as an isomer of **1**, which had the same molecular formula as determined by HRESIMS. The NMR data (Table 1) were almost superimposed to that of **1**, and the only noticeable differences were found in the tetramic acid part. For instance, C-3′ and C-4′, were upfield-shifted for 0.3 and 0.2 ppm, respectively, while C-5′ and C-6′ were downfield-shifted for 0.5 and 0.3 ppm, respectively. Meanwhile, H-5′ and H-6′a were upfield shifted (−0.03, −0.05 ppm), and H-6′b was shifted to downfield (+0.03 ppm). As the CD spectra of equisetin, phomasetin and their analogues were dominated by the stereochemistry of the decalin moiety [15], the similar CD spectra between **1** and **2** strongly indicated they shared the same absolute configuration for the decalin part. Hence, the discrepancy in NMR spectra could only be explained by a different configuration at C-5′, so they should be 5′-epimers of each other. Further evidence came from the chemical conversion experiment. Treatment of **2** with pyridine led to the epimerization at C-5′ (Scheme 1), and the epimerized product was found to be the same as **1** by HPLC (Figure 4). A scrutiny of the NMR data for the tetramic acid part (Table 2) revealed that compound **2** was much closer to phomasetin [16] than **1**, especially for the atoms around the chiral center (i.e., CH-5′ and CH_2_-6′). Thus, it was reasonable to deduce the same 5′*R* absolute configuration for **2**, as found in phomasetin. The structure of phomasetin was determined by spectroscopic analysis as well as by comparison with that of equisetin [15], and the latter compound was confirmed by total synthesis [17]. Hence, compound **1** has a 5′*S* stereochemistry as shown in Figure 1.

Interestingly, it was reported that the CD spectrum was qualitatively similar in epimers of equisetin and phomasetin [15], however, noticeable differences were found in the region of 226–260 nm for **1** and **2** (Figure 3). It is of note that CJ-21,058 [18] was found to possess the same planar structure as **1** and **2**, however, the configuration of the former compound had not been determined.

### 2.2. Larvicidal Activities

The isolated metabolites were evaluated for their inhibition against the fourth-instar larvae of the mosquito *Aedes aegypti*. Both **1** and **2** exhibited strong inhibition with the median lethal dose (LC_50_) values of 5.93 and 10.31 µg/mL, respectively (Table 3). This was comparable to the positive control, rotenone (LC_50_ = 3.49 µg/mL). It seems that the configuration at C-5′ has strong correlation with the larvicidal activities, as the epimer of **1** (i.e., **2**) was two times less active than that of **1**.

Decalin-type tetramic acid metabolites were found to display diverse activities, such as HIV-1 integrase inhibiting activity [19], antibacterial activity [18,20,21,22], antimalarial activity [23] and anti-parasite activity against *Trichomonas vaginalis* [24]. Our research revealed these type of compounds also have larvicidal function.

*Hyalodendriella* sp. was an interesting producer of larvicidal metabolites. Previously, dibenzo-α–pyrones were identified from this fungus as potent inhibitors of *A. aegypti* larvae [10]. In the current study, a new structural type of larvicidal metabolites was identified from the same species. This highlighted the high potential of bioprospecting larvicides from the endophytic fungi.

## 3. Materials and Methods

### 3.1. General Experimental Procedures

The optical rotations were measured on a Rudolph Autopol IV automatic polarimeter (Rudolph Research Analytical, Hackettstown, NJ, USA). The ultraviolet (UV) spectra were scanned by a TU-1810 UV-VIS spectrophotometer (Beijing Persee General Instrument Co., Ltd., Beijing, China). Circular dichroism (CD) spectra were recorded on a JASCO J-810 CD spectrometer (JASCO Corp., Tokyo, Japan). High resolution electrospray ionization mass spectrometry (HRESIMS) spectra were recorded on a Bruker Apex IV FTMS instrument (Bruker Daltonics, Bremen, Germany). 1D and 2D NMR spectra were recorded on an Avance 400 NMR spectrometer (Bruker BioSpin, Zurich, Switzerland). Chemical shifts were expressed in *δ* (ppm) and referenced to the solvent residual peaks at *δ*_H_ 7.26/*δ*_C_ 77.0 for CDCl_3_ and coupling constants (*J*) in Hertz (Hz). Semi-preparative HPLC separation was carried out on an Lab Alliance instrument (Scientific Systems Inc., State College, PA, USA) equipped with a Series III pump and an UV detector (Mode 210) using a Prevail C_18_ column, 250 mm × 10 mm, 5 μm (GRACE Corporate, Columbia, Maryland, USA). HPLC-DAD analysis was carried out on a Shimadzu LC-20A instrument with an SPD-M20A photodiode array detector (Shimadzu Corp., Tokyo, Japan) and an analytic C_18_ column 250 mm × 4.6 mm i.d., 5 μm (Phenomenex Inc., Torrance, California, USA). Column chromatography was performed using silica gel, 200–300 mesh (Qingdao Marine Chemical Inc., Qingdao, China). Precoated silica gel GF-254 plates (Qingdao Marine Chemical Inc., Qingdao, China) were used for analytical TLC. Spots were visualized under UV light (254 or 356 nm) or by spraying with 10% H_2_SO_4_ in 95% EtOH followed by heating at 110 °C for 10 min.

### 3.2. Fungal Material, Fermentation and Extraction

*Hyalodendriella* sp. Ponipodef12 was isolated from the hybrid poplar of *Populus deltoides* × *P. nigra* as described previously [9]. The fungus was cultured on potato dextrose agar (PDA) medium at 25 °C for 10 days, and then several agar plugs were used to inoculate the potato dextrose broth (PDB, 100 mL) in a 250 mL flask. The cultivation was performed in a shaker at 150 rpm and 25 °C for 7 days to produce the seed culture, which was used to inoculate the rice media in 1 L Erlenmeyer flasks each containing 150 g of rice and 150 mL of distilled water. The scale-up fermentation was carried out using a total of 10.5 kg of rice under static conditions at room temperature in the dark. After 45 days, the cultures were harvested, dried and ground. The dry materials were extracted with MeOH by exhaustive maceration, followed by concentration under vacuum at 40 °C to afford a brown residue, which was suspended in water and partitioned successively with petroleum ether and EtOAc to give the corresponding fractions. The EtOAc extract (96.2 g) was obtained and used for further investigation.

### 3.3. Isolation of Compounds ***1*** and ***2***

The EtOAc extract was subjected to chromatography over silica gel eluting with petroleum ether-acetone (8:1, *v*/*v*) to obtain six fractions (Frs. A–F). Fraction B (8.0 g) was chromatographed over silica gel using a gradient of petroleum ether–EtOAc (from 100:1 to 0:100, *v*/*v*) as eluent to yield nine subfractions (Frs. B1–B9). Subfraction B3 was purified by semi-preparative HPLC using MeOH–H_2_O (85:15, *v*/*v*) as the mobile phase to afford **1** (8.0 mg) and **2** (5.0 mg).

Hyalodendrin A (**1**): Colorless amorphous powder. [α]^26^_D_ +200.1 (*c* 0.98, CHCl_3_); UV (MeOH) λ_max_ 205, 235, 294 nm; CD (*c* = 6.45 × 10^−4^ M, MeOH) λ (Δε) 224 (+1.85), 238 (+3.86), 258 (+2.14), 288 (+5.58) nm; ^1^H (CDCl_3_, 400 MHz) and ^13^C (CDCl_3_, 100 MHz) NMR data, see Table 1; HRESIMS *m*/*z* 388.24854 [M + H]^+^ (calcd for C_23_H_34_NO_4_, 388.24824).

Hyalodendrin B (**2**): Colorless amorphous powder. [α]^26^_D_ +100.3 (*c* 4.27, CHCl_3_); UV (MeOH) λ_max_ 205, 235, 294 nm; CD (*c* = 6.45 × 10^−4^ M, MeOH) λ (Δε) 206 (+15.30), 240 (+0.61), 292 (+3.73) nm; ^1^H (CDCl_3_, 400 MHz) and ^13^C (CDCl_3_, 100 MHz) NMR data, see Table 1; HRESIMS *m*/*z* 388.24880 [M + H]^+^ (calcd for C_23_H_34_NO_4_, 388.24824).

### 3.4. Larvicidal Activity Assay

Compounds **1** and **2** were tested for their larvicidal activity against the fourth-instar larvae of *Aedes aegypti* as described previously [8]. Rotenone was used as the positive control. The results from all replicates in the larvicidal bioassay were subjected to probit analysis using the SPSS Statistics v.20.0 to determine LC_50_ and LC_90_ values. Significant differences in LC_50_ and LC_90_ were based on non-overlap of the 95% confidence intervals (CL) [25].

## 4. Conclusions

In this study, two new decalin-type tetramic acid analogues namely hyalodendrins A (**1**) and B (**2**) were isolated from the endophytic fungus *Hyalodendriella* sp. Ponipodef12 obtained from the hybrid poplar of *Populus deltoides* × *P. nigra*. The structures of **1** and **2** were elucidated by analysis of the spectroscopic data, including NMR, HRMS and ECD, and by chemical conversion. Both **1** and **2** exhibited potent larvicidal activity against the fourth-instar larvae of *Aedes aegypti*, which were promising candidates to be developed as larvicidal agents.

## Figures and Tables

**Table 1:** ^1^H (400 MHz) and ^13^C (100 MHz) NMR data of **1** and **2** (CDCl_3_).

Position	1		2	
	*δ*_C_, Type	*δ*_H_, Multi. (*J* in Hz)	*δ*_C_, Type	*δ*_H_, Multi. (*J* in Hz)
1	199.0, C	-	199.2, C	-
2	49.4, C	-	49.5, C	-
3	49.4, CH	3.06, m	49.3, CH	3.07, m
4	132.0, C	-	132.0, C	-
5	125.6, CH	5.15, s	125.6, CH	5.16, s
6	39.0, CH	1.82, m	39.2, CH	1.82, m
7	42.6, CH_2_	1.78, m 0.87, m	42.5, CH_2_	1.78, m 0.87, m
8	33.5, CH	1.50, m	33.5, CH	1.50, m
9	35.8, CH_2_	1.75, m 1.11, m	35.8, CH_2_	1.75, m 1.11, m
10	28.3, CH_2_	1.95, br. d (11.2) 1.02, m	28.2, CH_2_	1.96, br. d (11.9) 1.02, m
11	39.7, CH	1.66, m	39.7, CH	1.67, m
12	13.8, CH_3_	1.42, s	13.7, CH_3_	1.40, s
13	130.6, CH	5.11, m	130.6, CH	5.12, m
14	127.7, CH	5.23, dq (15.4, 6.0)	127.5, CH	5.24, m
15	17.8, CH_3_	1.53, d (6.0)	17.9, CH_3_	1.55, d (5.7)
16	22.2, CH_3_	1.60, s	22.2, CH_3_	1.59, s
17	22.5, CH_3_	0.91, d (6.7)	22.5, CH_3_	0.91, d (6.4)
2′	177.1, C	-	177.2, C	-
3′	100.2, C	-	99.9, C	-
4′	190.7, C	-	190.5, C	-
5′	66.2, CH	3.65, br. t (4.4)	66.7, CH	3.62, t (4.4)
6′	60.2, CH_2_	4.06, br. dd (11.5, 3.7) 3.85, dd (11.3, 5.0)	60.5, CH_2_	4.01, dd (11.5, 3.7) 3.88, br.dd (11.0, 4.0)
7′	27.2, CH_3_	3.04, s	27.3, CH_3_	3.05, s
1-OH		17.26, br. s		-

**Table 2:** Comparison of the NMR data (CDCl_3_) for the tetramic acid moiety.

Position	*δ*_C_			*δ*_H_		
	1	2	Phomasetin *^a^*	1	2	Phomasetin *^a^*
1	199.0	199.2	199.2	-	-	-
2′	177.1	177.2	177.3	-	-	-
3′	100.2	99.9	100.1	-	-	-
4′	190.7	190.5	190.8	-	-	-
5′	66.2	66.7	66.9	3.65, br. t	3.62, t	3.60, brs
6′	60.2	60.5	60.6	4.06, br. dd 3.85, dd	4.01, dd 3.88, br. dd	3.99, m 3.86, m
7′	27.2	27.3	27.5	3.04, s	3.05, s	3.01, s

*^a^* adapted from Reference [16].

**Table 3:** Larvicidal activities of **1** and **2** against *Aedes aegypti*.

Compound	LC_50_ (μg/mL) (95% CL)	LC_90_ (μg/mL) (95% CL)	Slope ± SD	*x* ^2^
**1**	5.93 (4.72–7.35)	20.99 (15.49–33.28)	2.33 ± 0.30	6.54
**2**	10.31 (8.52–12.31)	28.56 (22.31–41.68)	2.90 ± 0.38	9.98
Rotenone*^a^*	3.49 (2.85–4.19)	10.36 (8.04–15.22)	2.71 ± 0.36	6.43

*^a^* Positive control.

## Supplementary Materials

Supplementary materials (Figures S1–S11) associated with this article, including UV, HRESIMS, as well as 1D and 2D NMR spectra of compounds **1** and **2**, can be found in the online version.
