# Supplementary material for: Hyalodendrins A and B, New Decalin-Type Tetramic Acid Larvicides from the Endophytic Fungus Hyalodendriella sp. Ponipodef12
Source: Molecules. 2019 Dec 27;25(1):114. doi: 10.3390/molecules25010114 (PMC6982915; doi:10.3390/molecules25010114)
Supplement: Supplementary file 1 [file molecules-25-00114-s001.pdf]

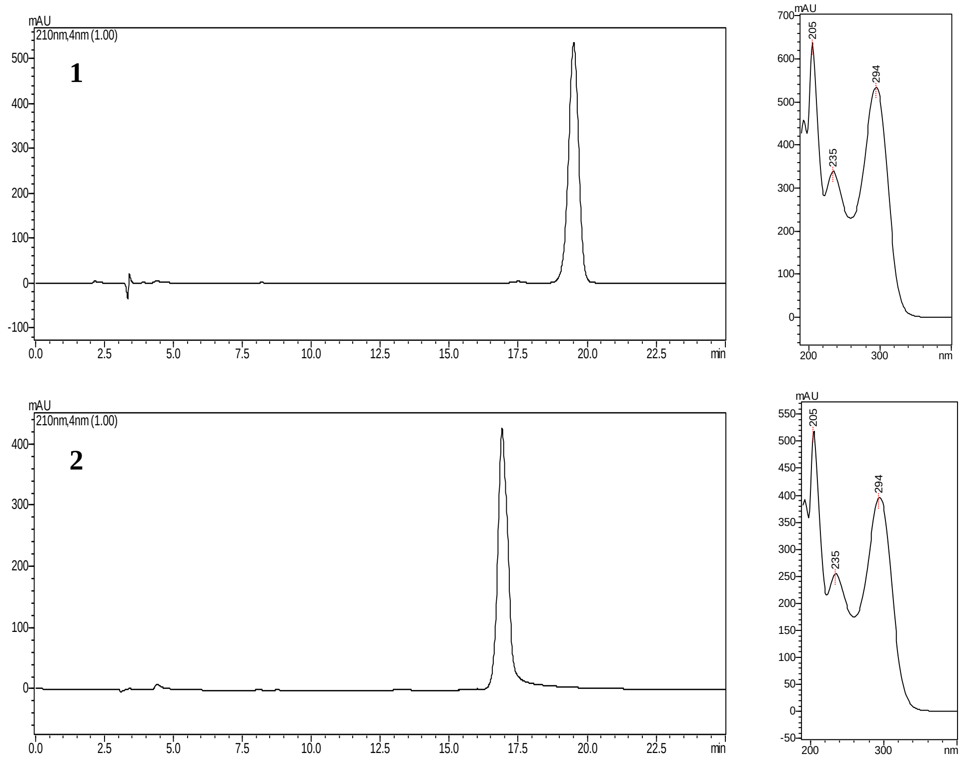

Figure S1. HPLC chromatograms (210 nm) and UV spectra of **1** and **2**.

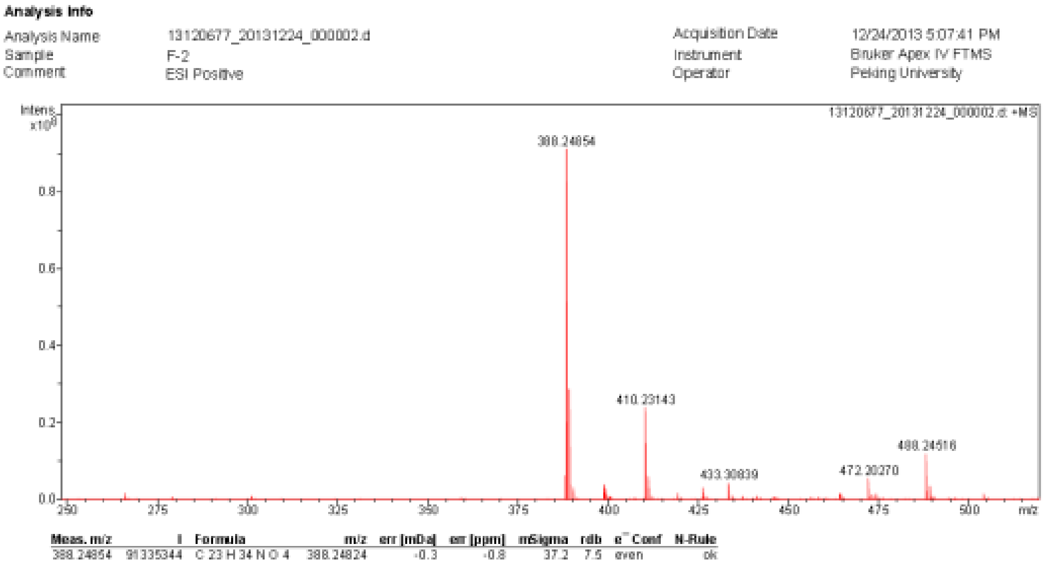

Figure S2. HRESIMS spectrum of **1**.

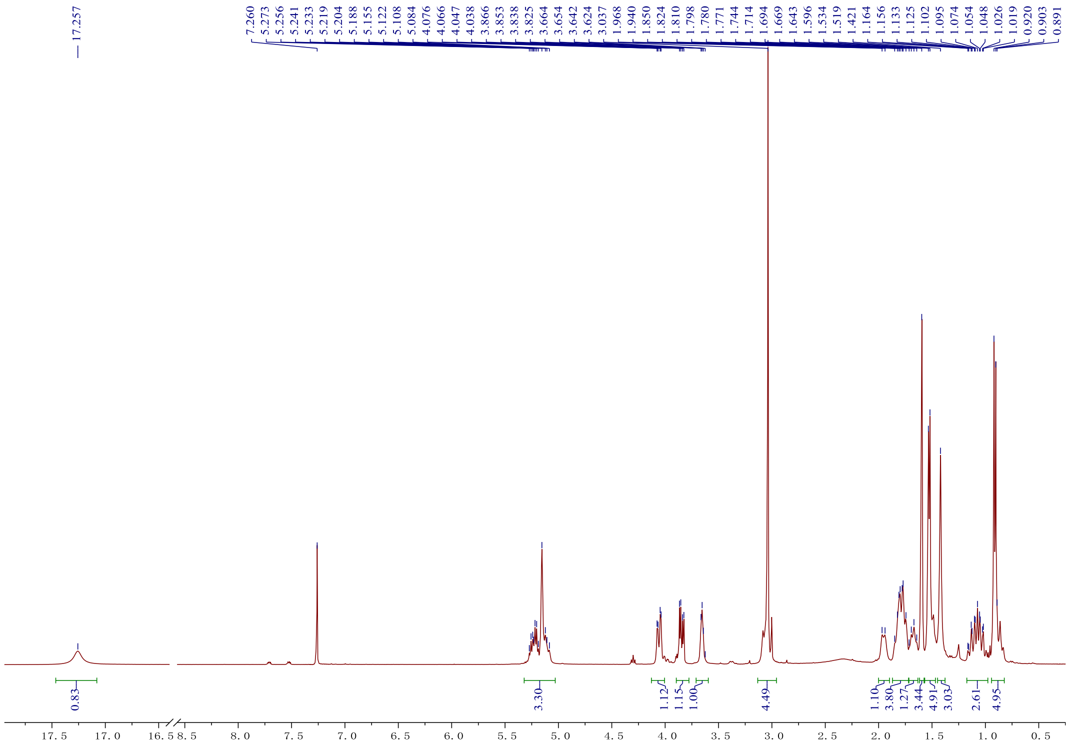

Figure S3. ^{1}H NMR spectrum of **1** ($CDCl_3$, 400 MHz).

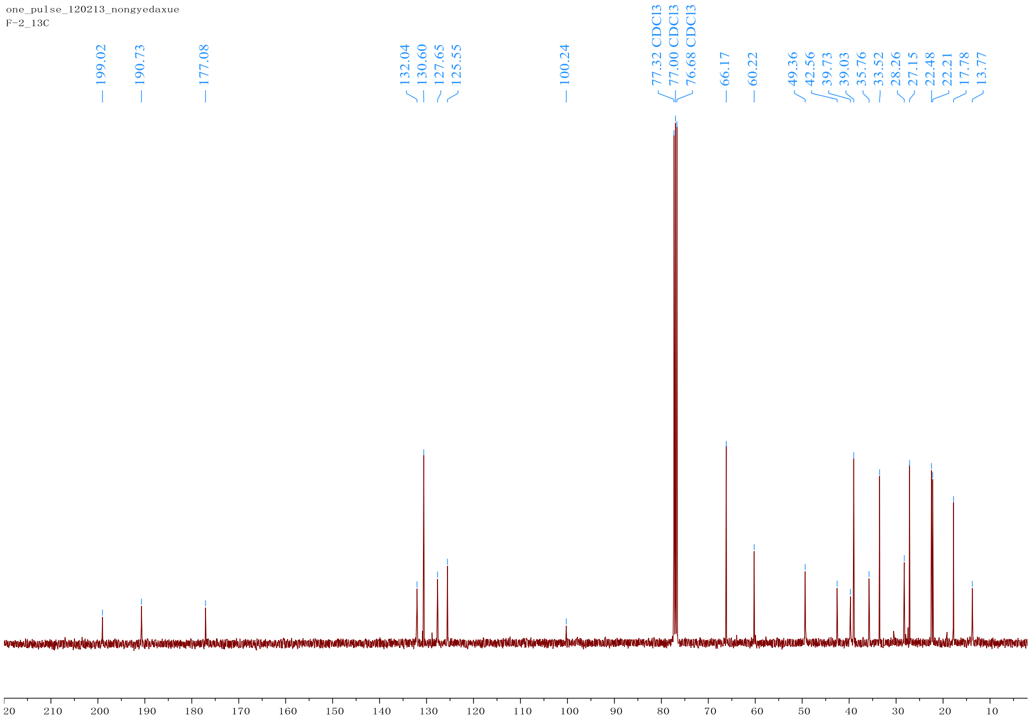

Figure S4. ^{13}C NMR spectrum of **1** ($CDCl_3$, 100 MHz).

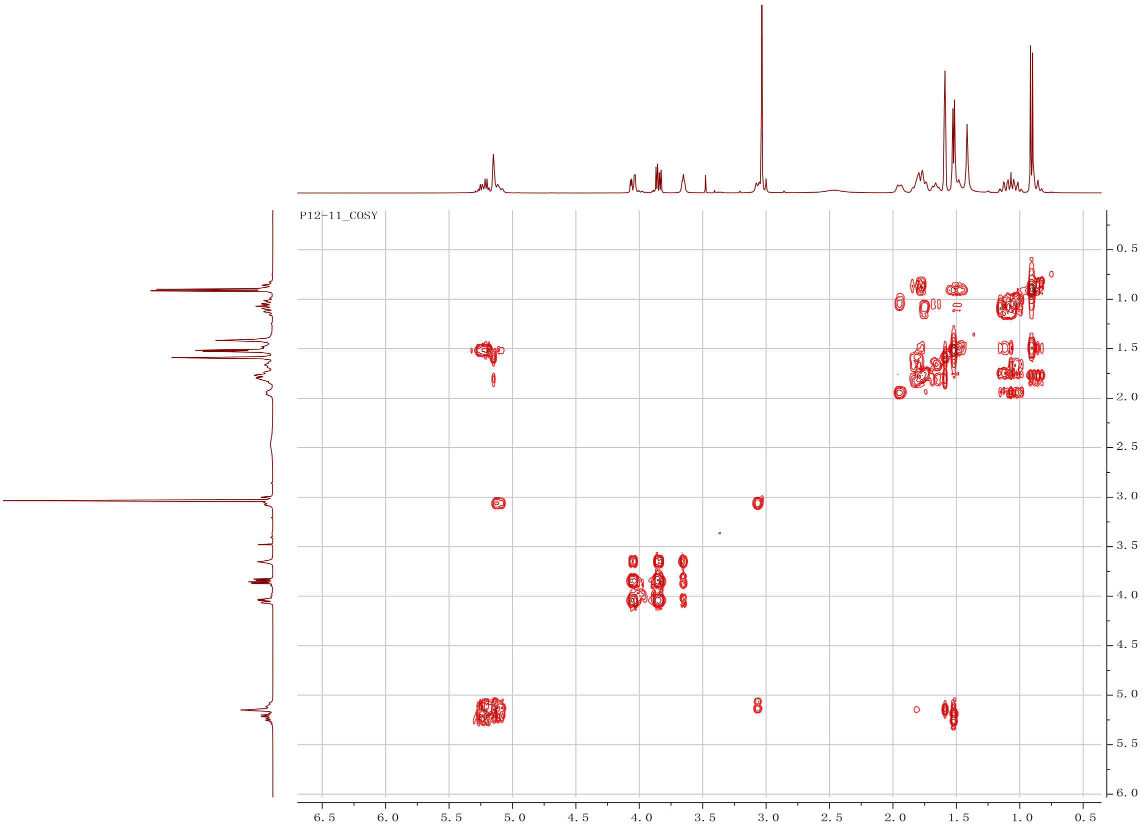

Figure S5. ^1H-^1H COSY spectrum of **1** ($CDCl_3$).

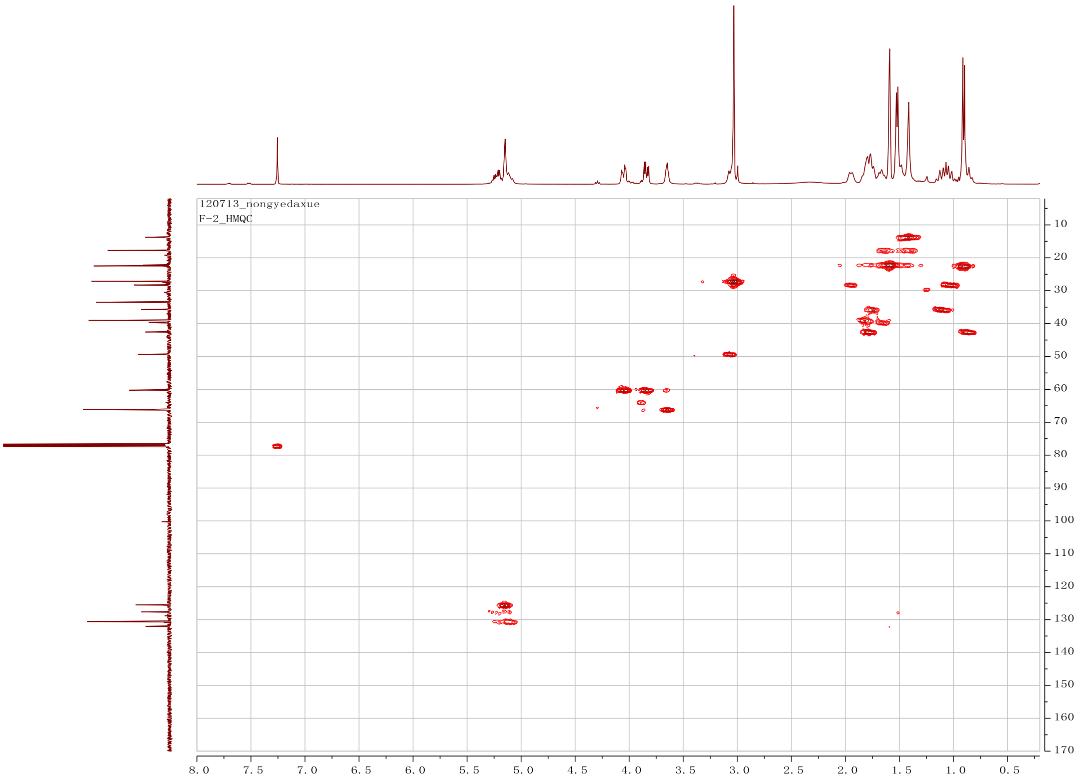

Figure S6. HSQC spectrum of **1** ($CDCl_3$).

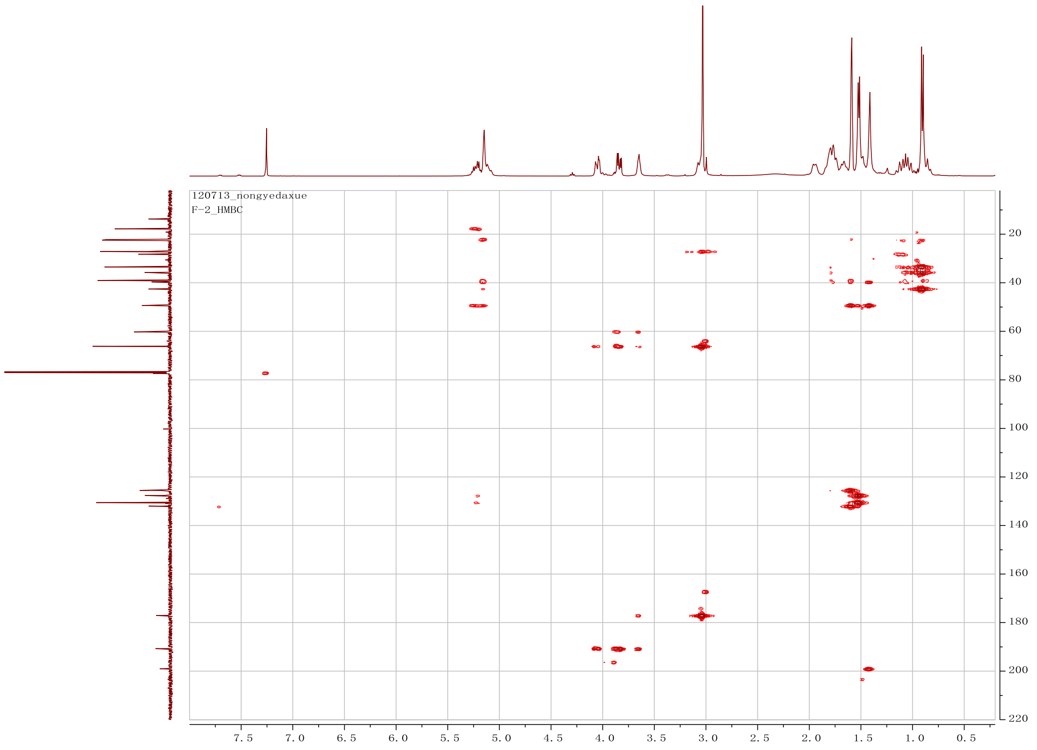

Figure S7. HMBC spectrum of **1** ($CDCl_3$).

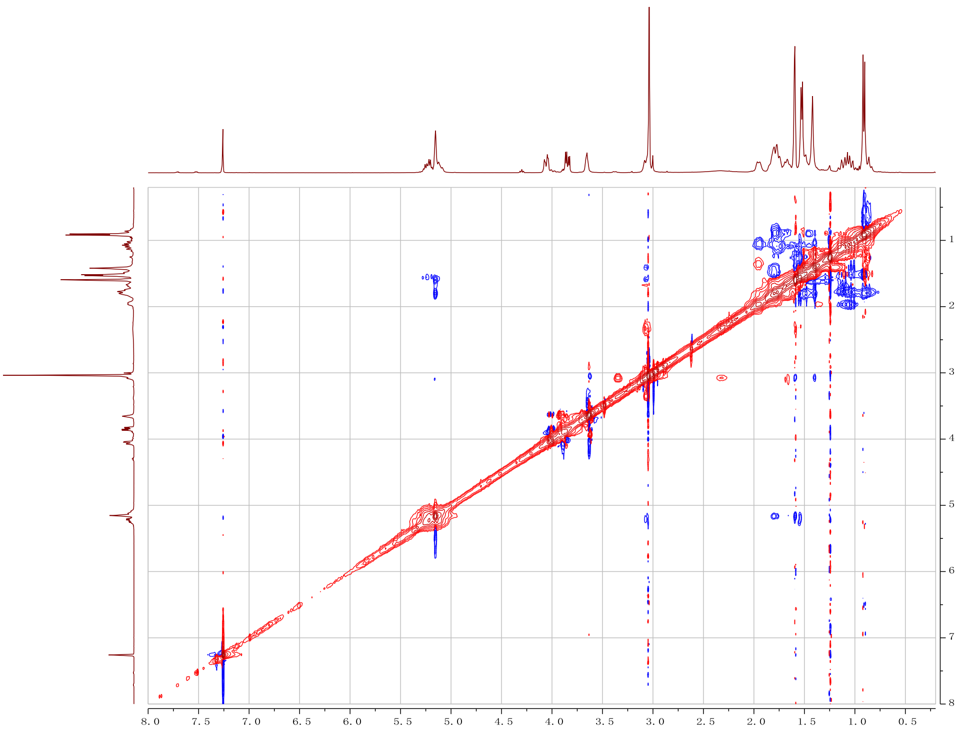

Figure S8. ROESY spectrum of **1** ($CDCl_3$).

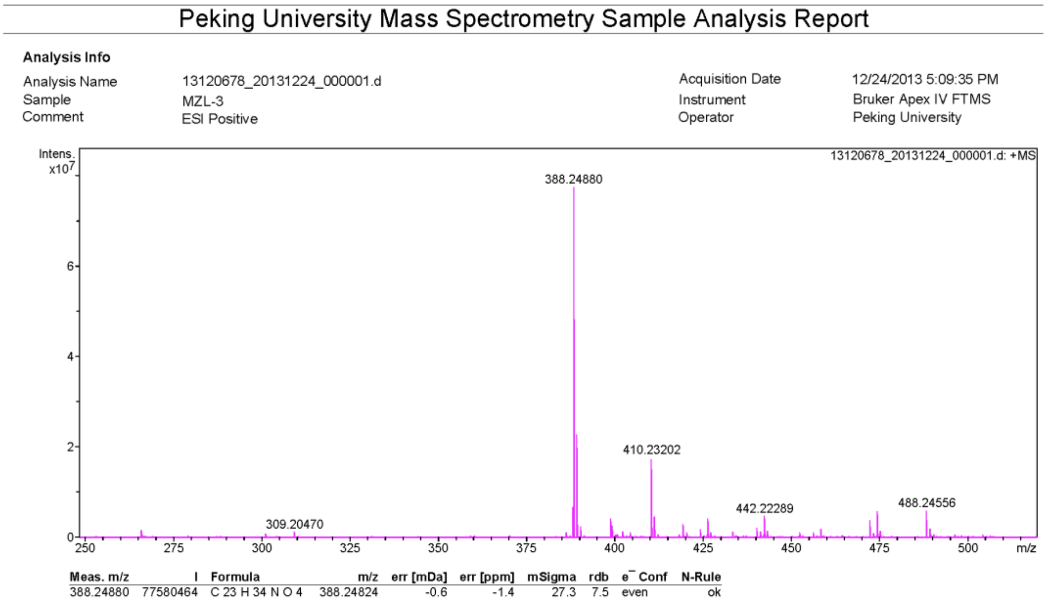

Figure S9. HRESIMS spectrum of **2**.

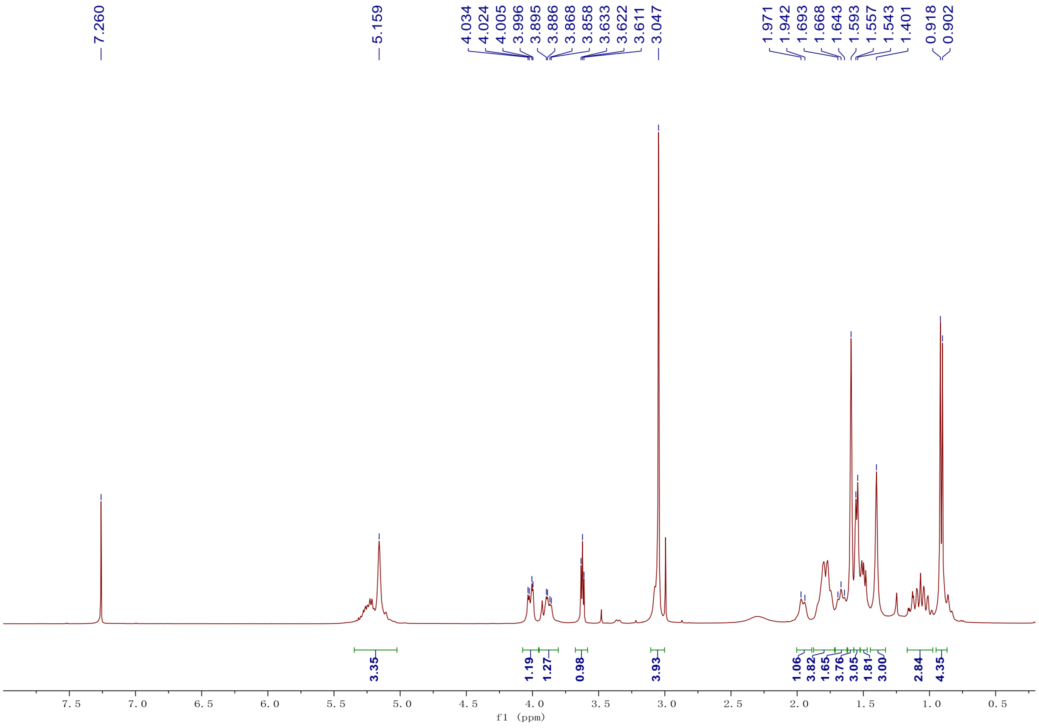

Figure S10. ^{1}H NMR spectrum of **2** ($CDCl_3$, 400 MHz).

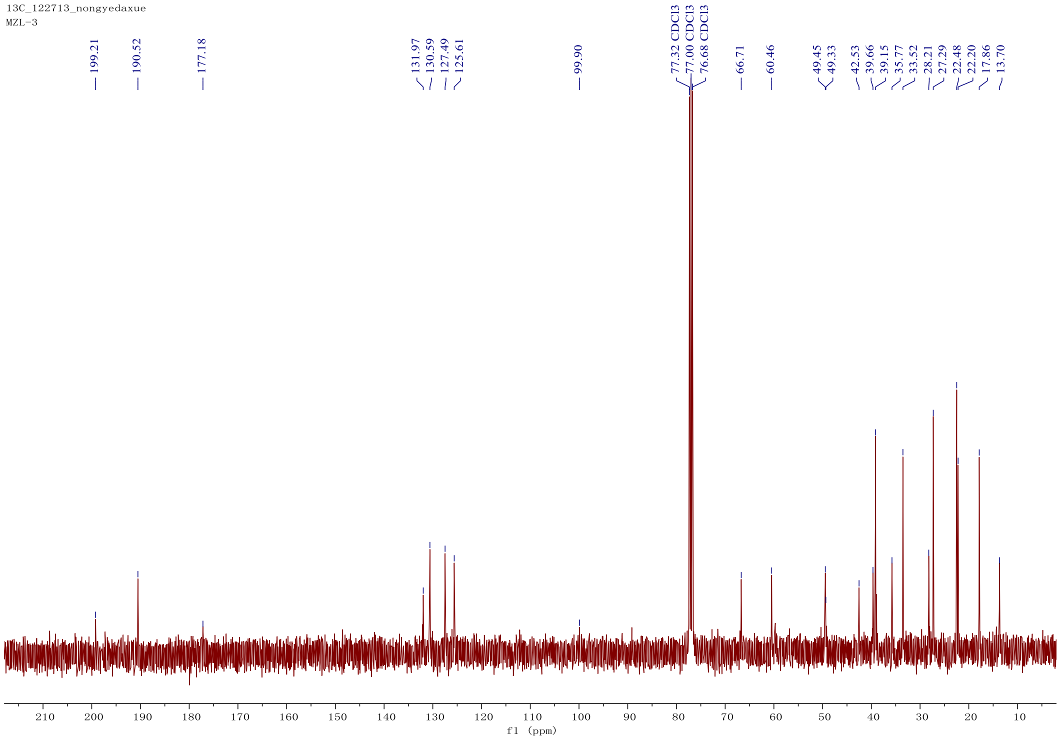

Figure S11. ^{13}C NMR spectrum of **2** ($CDCl_3$, 100 MHz).
